# HPV16-E2 protein modifies self-renewal and differentiation rate in progenitor cells of human immortalized keratinocytes

**DOI:** 10.1186/s12985-017-0736-2

**Published:** 2017-04-03

**Authors:** Victoria Domínguez-Catzín, Alicia-María Reveles-Espinoza, Janet Sánchez-Ramos, Raúl Cruz-Cadena, Diana Lemus-Hernández, Efraín Garrido

**Affiliations:** Laboratory of Research in Cancer Molecular and Cell Biology, Department of Genetics and Molecular Biology, CINVESTAV-IPN, Av. Instituto Politécnico Nacional 2508, Col. San Pedro Zacatenco, C.P. 07360 México D.F., Mexico

**Keywords:** HPV16-E2, Epithelial progenitor cells, HaCaT cells, Self-renewal, Epithelial differentiation, Immunophenotype, Stem cell markers

## Abstract

**Background:**

Cervical cancer is the fourth cause of death worldwide by cancer in women and is a disease associated to persistent infection with human papillomavirus (HPV), particularly from two high-risk types HPV16 and 18. The virus initiates its replicative cycle infecting cells located in the basal layer of the epithelium, where a small population of epithelial stem cells is located performing important functions of renewal and maintenance of the tissue. Viral E2 gene is one of the first expressed after infection and plays relevant roles in the replicative cycle of the virus, modifying fundamental processes in the infected cells. Thus, the aim of the present study was to demonstrate the presence of hierarchic subpopulations in HaCaT cell line and evaluate the effect of HPV16-E2 expression, on their biological processes.

**Methods:**

HaCaT-HPV16-E2 cells were generated by transduction of HaCaT cell line with a lentiviral vector. The α6-integrin-CD71 expression profile was established by immunostaining and flow cytometric analysis. After sorting, cell subpopulations were analyzed in biological assays for self-renewal, clonogenicity and expression of stemness factors (RT-qPCR).

**Results:**

We identified in HaCaT cell line three different subpopulations that correspond to early differentiated cells (α6-integrin^dim^), transitory amplifying cells (α6-integrin^bri^/CD71^bri^) and progenitor cells (α6-integrin^bri^/CD71^dim^). The last subpopulation showed stem cell characteristics, such as self-renewal ability, clonogenicity and expression of the well-known stem cell factors *SOX2*, *OCT4* and *NANOG*, suggesting they are stem-like cells. Interestingly, the expression of HPV16-E2 in HaCaT cells changed its α6-integrin-CD71 immunophenotype modifying the relative abundance of the cell subpopulations, reducing significantly the percentage of α6-integrin^bri^/CD71^dim^ cells. Moreover, the expression of the stem cell markers was also modified, increasing the expression of *SOX2* and *NANOG*, but decreasing notably the expression of *OCT4*.

**Conclusions:**

Our data demonstrated the presence of a small subpopulation with epithelial “progenitor cells” characteristics in the HaCaT cell line, and that HPV16-E2 expression on these cells induces early differentiation.

**Electronic supplementary material:**

The online version of this article (doi:10.1186/s12985-017-0736-2) contains supplementary material, which is available to authorized users.

## Background

High risk papillomavirus (HPV16 and 18) have been widely studied due to its association with cervical cancer and tumors of other anogenital sites [1]. These are small un-envolved virus with a circularized double stranded DNA genome, containing eight open reading frames that are expressed either early (E1, E2, E4, E5, E6 and E7) or late (L1 and L2) during the infection, and a noncoding region known as long control region (LCR) which contains the viral early promoter, the enhancer and the origin of replication [2, 3].

The HPV life cycle is intimately linked to epithelial differentiation [1]. HPV normally infects dividing cells at the basal layer of the epithelium but completes its life cycle by amplifying progeny DNA genomes in the spinous layer and carrying out viral encapsidation in the uppermost epithelial layer named the granular layer [4].

One of the proteins that plays a crucial role in the papillomaviruses life cycle is the E2 protein. It possesses a DNA-binding domain and a transactivation domain that are linked by a serine-arginine-rich hinge region [5]. E2 as a homodimer binds the cognate sequences ACCGN4CGGT (E2-binding sites [E2BSs]) in the viral LCR, and different experimental systems have shown that it regulates the viral E6 and E7 oncogenes transcription, acting as an activator or as a repressor depending on the protein levels and the specific sites contacted [6, 7]. However, the evidence points to a repressive function of E2 in controlling the viral early promoter [8]. On the other hand, another important role of E2 is to participate as an auxiliary replication factor, since at the origin of DNA replication, E2 interacts with and loads the HPV E1 replication helicase, recruiting then the cellular DNA replication machinery [8–11]. E2 also has an crucial role in segregation of viral episomal genomes during the division of infected cells by interacting with chromatin adapter proteins that tether them to host mitotic chromosomes [8].

In addition, possibly by an indirect mechanism involving its interaction with several cellular proteins, the expression of E2 also has an impact on the cellular transcription profile, affecting the expression of genes involved in key processes such as proliferation, differentiation, apoptosis and senescence [12–20].

The biological effects of E2 on mammalian cells have been generally studied using transiently transfected cancer cell lines. Early studies in HPV-positive cervical carcinoma derived cell lines [21], indicated that transient overexpression of E2 induces apoptosis in these cells due to repression of endogenous E6/E7 expression [22–27]. However, also in HPV negative cells, E2 overexpression induces apoptosis by both transactivation independent and mediated through activation of caspase 8 [28, 29]. It has been also observed that sustained expression of E2 in HPV positive cells, induces a prolonged growth arrest and induces irreversible senescence [30–33], probably as a default pathway for cells which exit the cell cycle, since most of these cells are incapable of terminal differentiation.

Then, the observed effects of E2 on the processes of apoptosis and senescence could be related to its role in differentiation, since it is well known that a critical step to start this process is an arrest in the cell cycle followed by the expression of genes involved in early differentiation. If these genes are not correctly expressed, cells could keep in an irreversible senescence status and progress later to apoptosis or immortalization programs [34, 35].

Considering the technical difficulty to establish a convenient cellular stratification system to follow step by step the progress of the infection, most of the studies on the effects of HPV gene expression on epithelial differentiation process have been performed on carcinoma derived cell lines [36–38], or immortalized keratinocytes from several tissues [39–41].

In fact, the induction of cell differentiation has been demonstrated in HPV16-E2 stably transfected HaCaT cells, showing a typical differentiated morphology, cell elongation and multilayer colonies growth, besides the expression of the classical keratinocytes differentiation markers involucrin, filagrin and cytokeratins 1 and 10 [34].

However, since E2 is one of the first expressed genes in the epithelial basal layer during infection [42, 43], the understanding of its effects on early differentiation must be studied on cellular models that mimic this layer in the epithelium, considering the cellular subpopulations that constitute it, including epithelial stem cells.

Different strategies have been used to identify these cells from epithelial derived transformed cell lines and keratinocytes primary cultures, such as the expression of the membrane molecules desmoglein-3 and β1-integrin, the nuclear presence of p63, or metabolic characteristics of these cells, such as the over-expression of aldehyde-dehydrogenase enzyme (ALDH) or the ATP-binding cassette subfamily G (ABCG) transporter pump [44–47]. Nevertheless, the simultaneous detection of α6-integrin (CD49f) and the transferrin receptor CD71 levels has demonstrated in a very confident form a hierarchical organization in keratinocytes primary cultures with the presence of three cellular subpopulations [48].

In the present work, we have taken advantage of the α6-integrin and CD71 detection and cell separation described by Schluter et al. [48], and demonstrated that HaCaT cell line derived from spontaneously immortalized keratinocytes, where HPV genomes are absent and have been extensively used to simulate epithelial tissues, possess cellular subpopulations with similar characteristics to those from the stratified epithelial basal layer, corresponding to: cells in an early differentiation process α6-integrin^dim^ (8.16 ± 0.52%), transitory amplifying cells α6-integrin^bri^/CD71^bri^ (87.27 ± 1.21%) and progenitor cells α6-integrin^bri^/CD71^dim^ (1.16 ± 0.08%) . This latter subpopulation expressed high mRNA levels of *SOX2*, *NANOG* and *OCT4* factors, a high self-renewal activity and a high proportion of holoclones formation in clonogenic assays, all of them characteristics of epithelial stem cells.

Besides, we demonstrated that HPV16-E2 expression modifies the relative abundance of these subpopulations, favoring the enrichment of the early differentiated subpopulation in a comparable way than the differentiation processes produced by the induction with retinoic acid (RA) or calcium chloride (CaCl_2_) in these cells.

## Methods

### Cell cultures

HEK293-FT cells from ATCC and HaCaT cells (a generous gift from Dr. Norbert Fusenig) were grown in culture dishes in Dulbecco’s modified Eagle’s medium (DMEM, Invitrogen, CA, USA) supplemented with 10% fetal bovine serum (FBS, Gibco, NY, USA), L-glutamine (2 mM), sodium pyruvate (1 mM), penicillin (50 U/ml), and streptomycin (50 μg/ml). Both cell lines were incubated in a humidified atmosphere with 5% CO_2_ at 37 °C and maintained in exponential growth phase.

### Lentiviral generation

A lentiviral system containing a cassette for puromycin selection and the transgene expression controlled by the promoter for the elongation factor 1-α (EF1-α), was used in this work. The E2 gene from HPV16 was amplified by PCR with the forward (Fw) primer 5′ ATTCCGAATTCATGGAGACTCT 3′ and the reverse (Rev) primer 5′ TTCGGGATCCTCATATAGACAT 3′, using as a template the plasmid pcDNA3-E2. The corresponding amplicon was cloned in the pSin-EF2-Pur plasmid (Addgene, MA, USA) using the EcoRI and BamHI restriction sites, generating the vector pSin-EF2-E216-Pur. A pSin-EF2-Vac-Pur vector was also built, incorporating the EcoRI-BamHI fragment from the pSin-EF2-Pur plasmid. This vector pSin-EF2-Vac-Pur allowed us to generate a lentivirus that does not contain expression cassette, denominated Lenti-Vac. Lentivirus were generated by co-transfection of the corresponding pSin-EF2-X-Pur with pMD2.G and psPAX2 plasmids into packing HEK293-FT cells using Lipofectamine Transfection Reagent (Invitrogen, CA, USA) during 24 h. After 48 h transfection, the supernatant from the cell cultures were ultracentrifugated (25,000 rpm in SW41 Ti rotor) for 2 h at 4 °C, to purify the lentiviral particles. The pellets were suspended in cold phosphate buffer saline (PBS) containing 0.01% bovine serum albumin (BSA) and stored at -70 °C.

### Lentiviral transduction

2.5 × 10^5^ HaCaT cells were seeded in DMEM with 10% SFB 24 h before the infection. The cell cultures were then incubated with 1 MOI (multiplicity of infection) of either HPV16-E2 lentivirus or Lenti-Vac for 24 h in DMEM with 10% SFB and polybrene (8 μg/ml), in order to allow virus adsorption. The viral stock was then removed away and 48 h post-infection the puromycin (Sigma-Aldrich, MO, USA) selection (0.45 μg/ml) was started.

### RNA extraction and gene expression analysis

Total RNA was extracted from cells using the TRIzol method, treated with RQ1 DNase (Promega, WI, USA) for 2 h at 37 ^o^C and 2 μg of RNA were reverse transcribed into cDNA using the enzyme M-MLV RT at 42 °C and Oligo-dT_15_ (Promega, WI, USA).

To determinate the transduction and the transgene expression, we amplify by PCR a 250 bp fragment of the HPV16-E2 gene, using primers Fw: 5′ TTGGGGATCCGTGTTTAGCAGCAACGAAGTAT 3′ and Rev: 5′ ATCCGAATTCTCAGTTAATCCGTCCTTTGTGTGAGCT 3′. HPV16-E2 expression in transduced cells was monitored daily.

To evaluate the mRNA expression of the stem cells markers we performed Real-Time PCR (qPCR) using the ABsolute qPCR SYBR Green Mix (Thermo Scientific, PA, USA) and an ABI StepOnePlus Real-Time PCR System, using the following primers: *SOX2* Fw: 5′ TCAGGAGTTGTCAAGGCAGAG 3′, Rev: 5′ AGAGGCAAACTGGAATCAGGA 3′; *NANOG* Fw: 5′ GCAATGGTGTGACGCAGAAG 3′, Rev: 5′ ATTGGAAGGTTCCCAGTCGG 3′; *OCT4* Fw: 5′ CTTCGCAAGCCCTCATTTCACC 3′, Rev: 5′ GGTCCGAGGATCAACCCAG 3′. As a control for endogenous constitutively expressed gene, we used β-actin Fw: 5′ GCGGGAAATCGTGCGTGACATT 3′, Rev: 5′ GATGGAGTTGAAGGTAGTTTCGTG 3′. Relative quantification values and the cycle thresholds (Cts) for the target amplicon and the endogenous control (β-actin) were determined for each sample (Total, α6-integrin^bri^/CD71^dim^ and Non-α6-integrin^bri^/CD71^dim^). The value for the target, normalized to the endogenous control for the samples, relative to the value in the total cells was then calculated with the formula ^ΔΔ^Ct [49].

### Immunoblotting analysis of proteins

Total cell lysates were prepared from HaCaT wild type (HaCaTwt) and HaCaT-HPV16-E2 using Bolen-modified RIPA buffer (20 mM MOPS-NaOH pH 7.0, 150 mM NaCl, 1% sodium deoxycholate, 1% Nonidet P-40, 1 mM EDTA, 0.1% SDS) and protease inhibitor cocktail (Roche Diagnostics, Basel, Switzerland). Total proteins (50 μg) were separated through SDS 10% polyacrilamide gels (SDS-PAGE), transferred to nitrocellulose membranes, and immunoblotted using either specific anti-HPV16-E2, anti-cytokeratin 14 (Santa Cruz Biotecnology Inc., CA, USA), anti-cytokeratin 10 (Abcam PLC, MA, USA) or anti-β-actin (a generous gift of Dr. Manuel Hernández) monoclonal antibodies. Secondary antibodies peroxidase conjugated AffiniPure goat anti-mouse IgG (H + L) or peroxidase conjugated AffiniPure goat anti-rabbit IgG (H + L) (Jackson ImmunoResearch Laboratories, PA, USA) were used. Immunoreaction was developed using the SuperSignal West Pico Chemiluminiscent Substrate (Thermo Scientific, IL, USA) and the proteins were quantified with Image J software (National Institutes of Health, MD, USA).

### Flow - cytometric analysis and cell sorting

HaCaT cell cultures (lentivirus transduced or wt) were washed twice in PBS, trypsinized, and suspended in ice-cold PBS containing 2% FBS/2% BSA and kept for 15 min at 4 °C. Cells were pelleted by centrifugation and suspended at 1 × 10^6^/100 μl in ice-cold PBS containing 1% BSA and then processed for single or double staining with PE-Cy5 mouse anti-human CD71 and PE rat anti-human α6 integrin (BD Biosciences, NJ, USA) during 45 min at 4 °C, using the appropriate negative controls to establish the compensation settings on the FACS. Cells were washed twice with cold PBS, suspended in PBS at 2–3 × 10^6^/ml, and kept at 4 °C until their flow-cytometric analysis. Cell sorting was performed using MoFlo XDP flow cytometer (Beckman Coulter, CA, USA).

### Self-renewal assay

α6-integrin^bri^/CD71^dim^ and Non-α6-integrin^bri^/CD71^dim^ HaCaTwt cells sorted after α6-integrin-CD71 staining, were seeded in six-wells plates at 4.5 × 10^3^ cells/well and cultured under standard conditions. After 10 days culture, cells were analyzed for cell surface phenotype (α6-integrin^bri^/CD71^dim^ and Non-α6-integrin^bri^/CD71^dim^) and sorted at subsequent passage for another cycle of 10 days to evaluate the self-renewal capacity.

### Colony heterogeneity assay

α6-integrin^bri^/CD71^dim^ and Non-α6-integrin^bri^/CD71^dim^ HaCaTwt cells sorted after α6-integrin-CD71 staining, were seeded in six-wells plates at 4.5 × 10^3^ cells/well and cultured under standard conditions. After 7 days culture, the morphology of the clones (classified as holoclones and paraclones) was observed and counted under an inverted light microscope evaluating the percentage of each type of clone.

### Differentiation analysis

HaCaTwt cells were seeded and cultured under standard conditions during 24 h. The cell cultures were then washed twice with PBS and incubated with complete media containing CaCl_2_ 5 mM or RA 1 μM (Sigma-Aldrich, MO, USA) during additional 5 days. Then cells were harvested and lysates prepared for the analysis of epithelial differentiation markers.

HaCaTwt and HaCaT-HPV16-E2 were treated with CaCl_2_ or RA as above indicated. After that, the cells were harvested and analyzed by flow cytometry for the α6-integrin-CD71 cell surface phenotype, in order to compare the differentiation status in HaCaTwt and HaCaT-HPV16-E2.

### Statistical analysis

Data were analyzed using GraphPad Prism^®^ v6.0 (GraphPad Software). Results represent the mean of at least three independent experiments; bar ± standard deviation (SD). An ANOVA test was used to determine the statistical significance of differences in values between two groups. Statistical significance was defined as *p* value <0.05.

## Results

### Identification of a “stem-like” subpopulation in HaCaT cell line

**Fig. 1:**
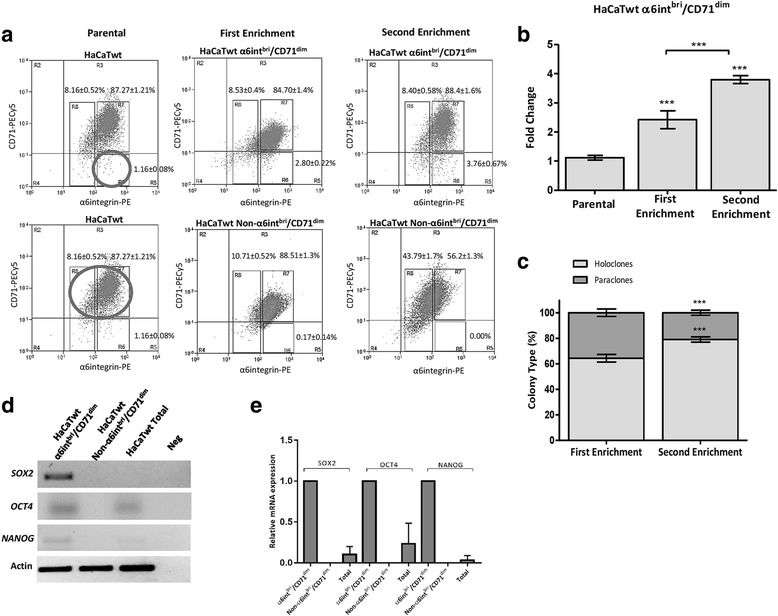
HaCaT cell line possesses a “progenitor” sub-population. **a** Flow cytometric analyses for α6-integrin and CD71 expression in HaCaT cell line exhibited three phenotypes: α6-integrin^dim^ (R8), α6-integrin^bri^/CD71^bri^ (R7) and α6-integrin^bri^/CD71^dim^ (R6). *Upper panels* correspond to enrichment assays sorting and reseeding only cells from the α6-integrin^bri^/CD71^dim^ subpopulation. *Lower panels* correspond to enrichment assays sorting and reseeding cells from a mix of α6-integrin^dim^ (R8) and α6-integrin^bri^/CD71^bri^ (R7) subpopulations. **b** Bars graph of two rounds enrichment of the α6-integrin^bri^/CD71^dim^ subpopulation in self-renewal assays. *Bars* represent the mean ± SD of three independent assays (*p* < 0.05). **c** Bars graph of the clonogenic assays from the α6-integrin^bri^/CD71^dim^ subpopulation seeded after each round of enrichment. *Bars* represent the mean ± SD of three independent assays (*p* < 0.05). **d** RT-PCR analyses for the expression of stem cell markers (*SOX2*, *OCT4* and *NANOG*) in α6-integrin^bri^/CD71^dim^ subpopulation, Non-α6-integrin^bri^/CD71^dim^ subpopulation and total population in HaCaT cell line. **e** RT-qPCR analyses for the expression of *SOX2*, *OCT4* and *NANOG* in total HaCaTwt cells and after sorting in α6-integrin^bri^/CD71^dim^ and Non-α6-integrin^bri^/CD71^dim^ subpopulations. Values are expressed as the difference in ^ΔΔ^Ct and expression of a housekeeping gene (β-actin). Relative expression of three separated assays expressed as the mean ± SD, *p* < 0.05, is presented. All images shown are representative of at least three independent experiments

To demonstrate the presence of a “progenitor” subpopulation in HaCaT cell line, we analyzed the cells using the technique described for Schluter et al. [48]. Representative results from the flow-cytometric analyses are shown in Fig. 1 panel a. The HaCaT cell line in our culture conditions presented a profile of three subpopulations with different immunophenothypes: α6-integrin^dim^ (8.16 ± 0.52%) (R8) that correspond to “early differentiated cells”, α6-integrin^bri^/CD71^bri^ (87.27 ± 1.21%) (R7) corresponding to “transitory amplifying cells” and α6-integrin^bri^/CD71^dim^ as a minor subpopulation (1.16 ± 0.08%) (R6) apparently corresponding to “progenitor” cells. To corroborate the biological characteristics of these cellular subpopulations, we performed a self-renewal assay sorting the cells after anti-α6-integrin-CD71 staining by flow cytometry and reseeding in separated wells cells from the α6-integrin^bri^/CD71^dim^ subpopulation and a mix of cells from the α6-integrin^bri^/CD71^bri^ and α6-integrin^dim^ subpopulations (Non-α6-integrin^bri^/CD71^dim^), and evaluated their capacity to regenerate the normal immunophenotypes of the cultures, enrichment ability and colony morphology in clonogenic assays.

α6-integrin^bri^/CD71^dim^ restored the immunophenotype of the total cell population in each cycle of sorting and reseeding, and after two rounds the percentage of this subpopulation increased from 2.8 ± 0.54% to 3.9 ± 0.33% (Fig. 1, panels a and b). The reseeding of the Non-α6-integrin^bri^/CD71^dim^ was unable to restore the immunophenotype of the total population, generating only Non-α6-integrin^bri^/CD71^dim^ (Fig. 1 panel a and Additional file 1: Table S1). Moreover, in the clonogenic assay the percentage of holoclones derived from the α6-integrin^bri^/CD71^dim^ after sorting and reseeding was considerably higher than those with paraclone morphology in both enrichment assays (62 ± 2% vs 38 ± 3.5% and 68 ± 5.5% vs 32 ± 3.5%) (Fig. 1, panel c), while as expected for more differentiated cells, the clone morphology observed after reseeding the Non-α6-integrin^bri^/CD71^dim^, was mostly paraclones (2% vs 98 ± 1% and 0% vs 100%). Finally, the mRNA evaluation of three of the most important stemness factors (*SOX2*, *NANOG* and *OCT4*) in the different subpopulations isolated by sorting after anti-α6-integrin-CD71 staining and flow-cytometry (Fig. 1, panels d and e), indicated that these genes are expressed in high levels in the α6-integrin^bri^/CD71^dim^ subpopulation but not in Non-α6-integrin^bri^/CD71^dim^. These results demonstrate that HaCaT cell line contains a small “progenitor” cell subpopulation.

### HPV16-E2 protein modifies α6-integrin-CD71 immunophenotype in HaCaT cell line

**Fig. 2:**
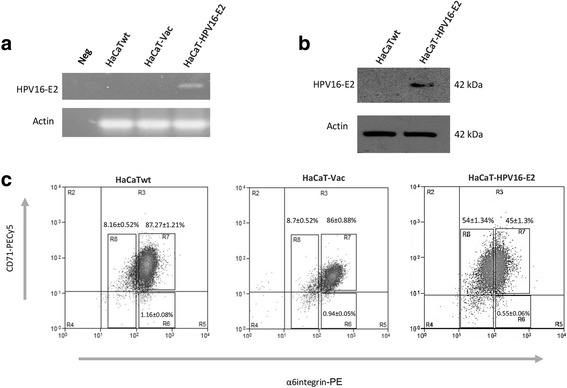
HPV16-E2 expression modifies the α6-integrin-CD71 subpopulations profile in HaCaT cell line. **a** RT-PCR analysis of lentivirus transduced HaCaT cells 5 days post-infection. HPV16-E2 is expressed only in transduced cells HaCaT-HPV16-E2. **b** Western blot analysis showing the expression of HPV16-E2 protein only in the transduced HaCaT-HPV16-E2 cells. **c** Representative flow cytometry analysis for the α6-integrin-CD71 subpopulations profile in HaCaT-HPV16-E2 cells. R6 decreases almost 50% while R8 increases at least 5 times in these cells. All images shown are representative of at least three independent experiments

It has been shown that HPV-E2 protein can modulate several cellular processes, including apoptosis, proliferation and cell differentiation. In order to evaluate the effect of the expression of this viral protein on α6-integrin-CD71 phenotype of HaCaT cell line, we used a lentiviral vector that efficiently transduce cells from epithelial origin, but also possess the EF1-α promoter that results in a high level of transcription of the incorporated transgene, even in quiescent cells. After transduction of HaCaT cell line cultures, with Lenti-HPV16-E2 or Lenti-Vac, we confirmed the expression of the transgene in the correspondent cultures HaCaT-HPV16-E2 and HaCaT-Vac 5 days post-infection, obtaining mRNA and analyzing by RT-PCR (Fig. 2, panel a). Additionally, we obtained total protein extracts and determined the presence of HPV16-E2 protein by SDS-PAGE and western blot, using commercial antibodies (Fig. 2, panel b). As expected, HPV16-E2 mRNA and protein was only detected in the HaCaT-HPV16-E2 transduced cell lines.

The flow-cytometric analysis for immunophenotype of α6-integrin and CD71 staining of the transduced HaCaT cultures (Fig. 2, panel c) showed evident changes in the relative abundance of the three subpopulations that constitutes this cell line, when HPV16-E2 is expressed. We observed a clear increase in the early differentiated cells (α6-integrin^dim^) passing from 8.16 ± 0.52% to 54 ± 1.34%, while the stem-like subpopulation (α6-integrin^bri^/CD71^dim^) decreased from 1.16 ± 0.08% to 0.55 ± 0.06%, suggesting that the expression of this viral gene stimulates the early differentiation in HaCaT cell line. To discriminate a possible effect of the lentiviral vector on the α6-integrin-CD71 phenotype in this cell line, we also compared this phenotype of Lenti-Vac infected cells against that one from non-infected cells, observing that infection with this control virus does not change the subpopulations profile of HaCaT cell line.

### HPV16-E2 protein modifies the expression of “stemness” factors in HaCaT cell line

**Fig. 3:**
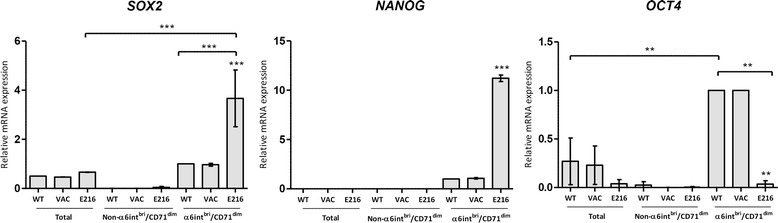
HPV16-E2 expression alters the level of stem cell markers in HaCaT cells. RT-qPCR analyses for expression of *SOX2*, *NANOG and OCT4* in HaCaTwt, HaCaT-Vac and HaCaT-HPV16-E2 total cell population and after sorting in α6-integrin^bri^/CD71^dim^ and Non-α6-integrin^bri^/CD71^dim^ subpopulations. Values are expressed as the difference in ^ΔΔ^Ct compared to non-infected cells and expression of a housekeeping gene (β-actin). Relative expression of three separated assays expressed as the mean ± SD, *p* < 0.05, is presented

Considering the clear reduction (almost 50%) in the relative abundance of the α6-integrin^bri^/CD71^dim^ subpopulation in the HaCaT cell line induced by HPV16-E2 expression, we evaluated the effect of the expression of this viral protein on the mRNA level of several key stemness factors. HaCaT cells were either transduced with Lenti-HPV16-E2 or Lenti-Vac, after 5 days were stained with anti-α6 integrin-CD71 antibodies and isolated by flow-cytometric sorting the α6-integrin^bri^/CD71^dim^ and Non-α6-integrin^bri^/CD71^dim^ subpopulations from HaCaT-HPV16-E2, HaCaT-Vac and HaCaTwt. mRNA from each subpopulation was obtained and the expression of *SOX2*, *OCT4* and *NANOG* genes analyzed by RT-qPCR (Fig. 3). As we expected, the Lenti-Vac transduction does not modified the relative expression of *SOX2*, *NANOG* or *OCT4* in neither of the HaCaT cell subpopulations; nevertheless, in α6-integrin^bri^/CD71^dim^ subpopulation from HaCaT cells expressing HPV16-E2 the mRNA level of *SOX2* and *NANOG* was increased dramatically (5 and 10 times respectively), compared with the level observed in the α6-integrin^bri^/CD71^dim^ subpopulation in HaCaTwt cells. Interestingly, the effect of HPV16-E2 on *OCT4* expression was in the opposite way, since the mRNA levels detected for this factor in the α6-integrin^bri^/CD71^dim^ subpopulation from HaCaT-HPV16-E2 were considerably lower (0.2 times) than the observed in the HaCaTwt α6-integrin^bri^/CD71^dim^ subpopulation.

All these changes in the expression of stemness factors induced by E2 in the α6-integrin^bri^/CD71^dim^ subpopulation, suggest that this viral protein in HaCaT could be regulating the fate of the cells either to keeping in a stemness status or the exit of these “progenitor” cells to a more differentiated status.

### HPV16-E2 protein induces early differentiation in HaCaT cell line

**Fig. 4:**
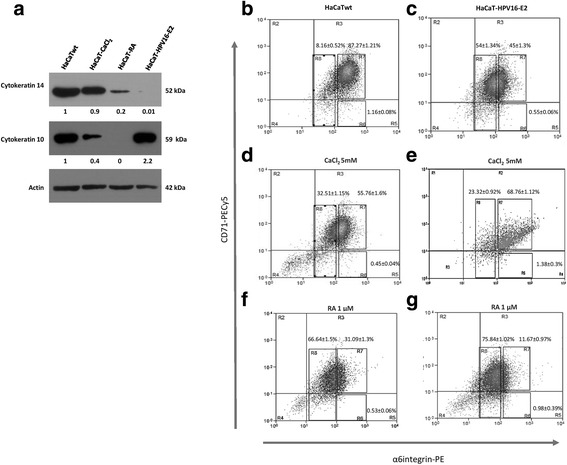
HPV16-E2 protein promotes differentiation in HaCaT cells in a comparable way than epithelial differentiation inducers. **a** Western blot analysis showing the expression of cytokeratin 10 and 14 proteins in non-stimulated HaCaTwt cells, 5 days 5 mM CaCl_2_ or 1 μM RA stimulated HaCaTwt cells, and HaCaT-HPV16-E2 cells. Numeric values below each lane are the quantitation results expressed as the ratio to non-stimulated HaCaTwt cells and normalized against β-actin. Flow - cytometric analysis. *Left panels* (**b**, **d** and **f**), HaCaTwt cells; *right panels* (**c**, **e** and **g**), HaCaT-HPV16-E2 cells. **b** and **c**, non-stimulated cells; **d** and **e**, CaCl_2_ stimulated cells; **f** and **g**, RA stimulated cells. All images shown are representative of at least three independent experiments

The increase observed in the α6-integrin^dim^ subpopulation in HaCaT-HPV16-E2 cells, strongly suggest that expression of the viral protein induces the cells to differentiation. Then we decided to confirm this, first analyzing the relative levels of cytokeratin 14, a protein expressed in all the constituent cells of the epithelial basal layer [50], and cytokeratin 10, a protein expressed *de novo* by early differentiated cells [50, 51]. Considering the well known effects of differentiation induced on HaCaT cells by CaCl_2_ and RA treatments, we exposed HaCaTwt cells to either 5 mM CaCl_2_ or 1 μM RA for 5 days and used as a reference to compare the expression level of both cytokeratins in HaCaT-HPV16-E2 cells, by western blot analysis. We observed in HaCaT-HPV16-E2 cells a considerable lower level of cytokeratin 14 and more than twice level of cytokeratin 10, compared to HaCaTwt (Fig. 4, panel a). This clearly demonstrates that HPV16-E2 protein promotes early differentiation in HaCaT cells. Although CaCl_2_ or RA treatments induced in HaCaTwt cells a decrease in the protein levels of cytokeratins 14 and 10, the diminution caused by RA was more significant provoking a reduction of 80% in cytokeratin 14 and 100% in cytokeratin 10 protein levels, while the decrease observed with CaCl_2_ treatment was 10 and 60% respectively (Fig. 4, panel a). These results indicate that both treatments induce cell differentiation in a faster way and probably by a different pathway than HPV16-E2, since the low level of cytokeratin 10 in exposed cells suggest they are in a later step in the cell differentiation process, as has been demonstrated in several reports [52, 53].

Having in mind that the effects of these differentiation inducers on the α6-integrin-CD71 subpopulations profile has not been reported, we exposed HaCaT cultures to either CaCl_2_ or RA as described above and analyzed the subpopulations profile obtained by flow cytometry from the α6-integrin-CD71 stained cells. We observed that both, CaCl_2_ and RA treatments induced significant changes in the α6-integrin-CD71 subpopulations profile in HaCaT cell line; however clear differences between them were observed suggesting that induction of differentiation with each one of these agents is not equivalent. The main difference observed was that while CaCl_2_ induced a considerable enrichment in the early differentiated cells (α6-integrin^dim^ subpopulation) (32.51 ± 1.15%) (Fig. 4, panel d), the enrichment induced by RA treatment in this subpopulation was even higher (66.64 ± 1.5%) (Fig. 4, panel f).

Considering that cells can naturally receive extracellular stimulus for differentiation independently of the HPV16-E2 expression, we exposed HaCaT-HPV16-E2 cells to either, CaCl_2_ or RA treatments for 5 days and evaluated the subpopulations profile obtained by flow cytometry from α6-integrin and CD71 stained cells. Interestingly the subpopulation profile generated was particular for each differentiation inducer agent used. In HaCaT-HPV16-E2, CaCl_2_ treatment induced a considerable increase in the relative abundance of the α6-integrin^bri^/CD71^dim^ subpopulation (0.55 ± 0.06% vs 1.38 ± 0.3%) (Fig. 4, panels c and e [R6]), reaching even higher amounts than the percentage of cells present in this subpopulation in HaCaTwt cultures (1.16 ± 0.08%) (Fig. 4, panel b). Furthermore, this treatment also induced an increase in the relative abundance of the α6-integrin^bri^/CD71^bri^ subpopulation, considered transitory amplifying cells (45 ± 1.3% vs 68.76 ± 1.12%). This suggests that CaCl_2_ treatment restricts HPV16-E2 expressing cells to a differentiation state still able to proliferate actively (Fig. 4, panels c and e [R7]).

By the other side, RA treatment induced in HaCaT-HPV16-E2 cells an even more marked effect on the differentiation status than on HaCaTwt cells, leading cells mostly to early differentiation, since the relative abundance of the α6-integrin^dim^ subpopulation observed was a little higher (66.64 ± 1.5% vs 75.84 ± 1.02%) (Fig. 4, panels f and g [R8]) in HaCaT-HPV16-E2 cells. In a similar way than the observed in CaCl_2_ treated HaCaT-HPV16-E2 cells, RA also increased visibly the relative abundance of the α6-integrin^bri^/CD71^dim^ subpopulation, compared with non-treated HaCaT-HPV16-E2 cells (0.55 ± 0.06% vs 0.98 ± 0.39%) (Fig. 4, panels c and g [R6]).

These results suggest that HPV16-E2 protein presence modifies cellular mechanism inducing the early differentiation of the α6-integrin^bri^/CD71^dim^ subpopulation in the HaCaT cell line.

## Discussion

HPV infection in the basal epithelial layer may occur in any cellular type that integrates this layer and then the expression of the early genes, E2 one of the most important in this virus, could have particular effects on each one of the constituent cell types. A limited number of cells in this epithelial layer, possess cell progenitor characteristics and this small subpopulation has been identified and isolated from primary cultures and transformed cell lines by using different experimental approaches such as detection of desmoglein-3, α6- and β1-integrins, CD71, the expression and nuclear presence of p63, or the metabolic activity of ALDH or ABCG transporters in these cells [44–47].

HaCaT cell line that derives from spontaneously immortalized keratinocytes has been widely used to simulate epithelial tissues [54, 55]. Using the method described by Schluter and cols. [48], in this work we demonstrated for the first time that cultures of the HaCaT cell line, similarly to the reported in keratinocytes primary cultures, contain cellular subpopulations, based on the identification and simultaneous detection of the membrane receptors α6-integrin and CD71, corresponding to: early differentiated cells (α6-integrin^dim^), transitory amplifying cells (α6-integrin^bri^/CD71^bri^) and progenitor cells (α6-integrin^bri^/CD71^dim^) (Fig. 1, panel a).

Self-renewal assays, clonogenicity and expression of the transcription factors *NANOG*, *SOX2* and *OCT4* have been currently used to demonstrate the stemness of putative progenitor cell populations, no matter the method used for its isolation, either based on metabolic characteristics or expression level of specific membrane proteins [45, 56–58].

The self-renewal capability observed in cells from the α6-integrin^bri^/CD71^dim^ subpopulation in HaCaT cells reported in this work, was similar to that reported in several works in the identification of both adult normal and cancer stem cells, where the isolated progenitor subpopulation was also able to reestablish the initial phenotype of the total population, increasing also the number of putative “progenitor” cells after each cycle of separation [46, 59, 60]. However, in a clear contrast with cancer stem cells, putative progenitor cell population in HaCaT was considerably lower and its enrichment after reseeding was not exponential.

The ability of cells from the α6-integrin^bri^/CD71^dim^ subpopulation to form in clonogenic assays mostly holoclones (Fig. 1, panel c), in a similar way than the reported in keratinocytes primary cultures [59], suggest also that cells from this subpopulation have properties of progenitor epithelial cells.

The presence of mRNA from transcription factors *SOX2*, *NANOG* and *OCT4* (Fig. 1, panels d and e), whose expression has been reported for progenitor cells in normal keratinocytes [56, 61], only in the α6-integrin^bri^/CD71^dim^ subpopulation of HaCaT cells, largely explains its self-renewal capability and the formation mainly of holoclones. In agreement with our results, a recent work reported the immunodetection of NANOG and OCT4 proteins in HaCaT cell line growing in monolayer conditions, despite in those particular culture conditions and unlike than the cancer stem cells evaluated in that work, HaCaT cells were not capable to form spheroids [45]. Taken together these results confirm that α6-integrin^bri^/CD71^dim^ subpopulation that constitutes approximately 1% of the total population in HaCaT cell line, is enriched in progenitor cells.

Although HaCaT is an immortalized cell line, it possesses particular characteristics that make it very attractive to be used as biological system, such as the absence of viral sequences in its genome, and its ability to respond to a variety of differentiation stimuli such as CaCl_2_, RA and cell-cell contact [62–64]. Our demonstration that this cell line possesses, in a low but constant proportion, a cellular subpopulation that express stemness markers, with self-renewal capability similar to the observed in normal epithelial stem cells, constitutes an additional property that must be exploited in this cell line. Then, HaCaT cell line would seem to be a very appropriate model to study the effects of viral gene expression on the three main subpopulations that constitute the epithelial basal layer and that can be target for the HPV infection.

We observed that expression of HPV16-E2 in HaCaT cells generated a significant change in the proportion of the three cellular subpopulations, possibly favoring the early differentiation, since the relative abundance of progenitor (α6-integrin^bri^/CD71^dim^) cells decreased notably, while cell subpopulation committed to differentiation (α6-integrin^bri^) increased (Fig. 2, panel c). These results confirm and complement the observed by Burns and cols., whom reported the expression of early differentiation markers in the total population of HaCaT cells expressing HPV16-E2 [34]. In the same way, previous reports from our research group in C-33A cells, indicated that the presence of HPV16-E2 modifies the gene expression profile affecting importantly several signaling pathways, such as integrins, WNT/β-catenin, RhoA and Notch [20], all of them relevant for proliferation and cell differentiation responses.

Although the stem like properties of the HaCaT-HPV16-E2 cells might not change drastically, some aspects of stemness, such as *SOX2*, *NANOG* and *OCT4* expression are altered. Then, changes in the relative abundance of the subpopulations could be just another effect of the expression of HPV16-E2, considering that our interpretation is based exclusively on the expression of α6-integrin and CD71. Interestingly the effect on the expression of the stemness factors is differential, since while for *SOX2* and *NANOG* the expression is higher in these cells, the opposite effect is observed for *OCT4* (Fig. 3), suggesting that characterization of the E2 cells may not be so straightforward.

The modification in the expression level of these stemness factors could be related to the ability of HPV16-E2 protein to physically interact with several transcription factors such as CBP/p300, SP1, TAF1 and BRD4 [33, 65–68], all of them involved in the regulation of crucial genes for proliferation and differentiation pathways, and also in the regulation of *SOX2*, *NANOG* and *OCT4* expression [56, 61]. As an example, it has been demonstrated that BRD4 is a very important piece in the regulation of *NANOG* gene expression [68], and the well known capability of HPV16-E2 to stabilize the binding of BRD4 to cellular promoters [69] could explain the overexpression observed in cells where HPV16-E2 is present. On the other side, it has been reported that SP1 factor binds directly to the *OCT4* promoter favoring its transcription [70]. In this way, the physical interaction of E2 protein with SP1 [71, 72] could prevent its union to several gene promoters, *OCT4* among them, explaining the low expression level of this factor observed in our assays.

The changes generated in the expression pattern of the stemness genes in the progenitor cells in HaCaT-HPV16-E2, suggest that the viral protein has a similar effect than the previously described for differentiation inducer agents [73–76]. Conditions previously reported for stimulation of differentiation in HaCaT cells with CaCl_2_ or RA [77, 78], caused a similar effect on the subpopulations profile to that observed in HaCaT cells expressing HPV16-E2 (Fig. 4, panels c, d and f), confirming that this viral protein by itself is capable to induce a differentiation process. Until our knowledge, the effect of these differentiation inducers agents on the cellular subpopulations constituents of this cell line, has not been reported, then the present work is pioneering in this respect.

RA treatment of HaCaT-HPV16-E2 cells generated the apparent acceleration in the differentiation process, since the majority of cells in the total population corresponded to those committed to early differentiation (Fig. 4, panel g). However, CaCl_2_ treatment generated a profile where almost 70% of the cells corresponded to the transitory amplifying subpopulation. This means that in cells expressing HPV16-E2, the stimulus induces initially an accelerated proliferation process, and then cell differentiation (Fig. 4, panel e). This behavior could be explained by an increase in the activity of the cip/kip family member p21, since it has been demonstrated that this gene is transcriptionally activated by HPV16-E2 protein [33, 71, 79], and also by the cascade S100C/A11 directly induced by CaCl_2_, leading the cells to hyperproliferation [77]. The same observed behavior could be also related to the expression or the absence of different regulators of the epidermal differentiation, such as the insulin growth factor binding protein 3 (IGFBP-3), which is characteristically expressed in cells from the epithelial basal layer and the repression of this factor by CaCl_2_ stimulus, leads cells to proliferation [80]. Interestingly, the effect of either CaCl_2_ or RA treatment on the relative abundance of the progenitor subpopulation in HaCaT-HPV16-E2 cells was radically different than the observed in HaCaTwt cells, inducing an increase and reaching 0.98% for RA and 1.38% for CaCl_2_ induction (Fig. 4, panels d, e, f and g). This comportment generated by E2 expression could be due to both, the direct transcriptional regulation of genes involved in particular pathways, as well as to indirect mechanisms at epigenetic level, in genic regions that regulate cell fate decision to stemness or to differentiation.

Taken together these results suggest that the synergy between the differentiation stimulus that represents the expression of HPV16-E2 accompanied of a second differentiation stimulus such as CaCl_2_ or RA (HPV16-E2/CaCl_2_ and HPV16-E2/AR) can promote the exit of some of the “progenitor cells” to the next immediate higher differentiation level, while the remaining percentage of this subpopulation is apparently less receptive to the stimulus, giving to these cells the opportunity to stays in a stemness status. These effects could guarantee the continuation of the replicative cycle that depends of the epithelial differentiation, besides the preservation of a small amount of progenitor cells ensuring the viral persistence.

## Conclusions

In summary, we determined that HaCaT cell line possess three subpopulations that correspond to early differentiated, transitory amplifying and early progenitor cells. This latter population fully accomplishes the stem-like characteristics, such as enrichment, self-renewal, regeneration of the total population and expression of the stemness factors *NANOG*, *SOX2* and *OCT4*. The HPV16-E2 expression modifies the relative abundance of the subpopulations, and affects the expression of the stemness factors. These changes could be involved in the control of the exit from stemness to transitory amplified cells or early differentiated cells. Further research on the effects of HPV16-E2 expression and the differentiation pathways it regulates is needed.

## Additional file

Additional file 1: Table S1. Data from the self-renewal assays. HaCaTwt cells were α6-integrin-CD71 stained and analyzed by flow cytometry to establish the relative abundance of α6-integrin^bri^/CD71^dim^ (R6), α6-integrin^bri^/CD71^bri^ (R7) and α6-integrin^dim^ (R8) subpopulations (Parental). After sorting, α6-integrin^bri^/CD71^dim^ or Non-α6-integrin^bri^/CD71^dim^ cells were re-seeded in separated wells and grown for 10 days. Then, the immunophenotype was analyzed by flow cytometry (First enrichment). Again, α6-integrin^bri^/CD71^dim^ cells or Non-α6-integrin^bri^/CD71^dim^ cells were sort and re-seeded for 10 days and flow cytometer analyzed (Second enrichment). Data are presented as the average and SD from three self-renewal assays. (DOCX 13 kb)

## Abbreviations

°C: Degrees Celsius
ABCG: ATP binding cassette subfamily G
ALDH: Aldehyde-dehydrogenase enzyme
bp: Base pairs
bri: Bright
BSA: Bovine serum albumin
CaCl_2_: Calcium chloride
cDNA: Complementary DNA
Cts: Cycle thresholds
DMEM: Dulbecco’s modified Eagle’s medium
DNA: Deoxyribonucleic acid
EDTA: Ethylenediaminetetraacetic acid
EF1-α: Elongation factor 1-α
FACS: Fluorescence activated cell sorting
FBS: Fetal bovine serum
Fig.: Figure
Fw: Forward
HaCaTwt: HaCaT wild type
HPV: Human papillomavirus
h: Hours
IGFBP-3: Insulin growth factor binding protein 3
LCR: Long control region
Lenti-HPV16-E2: HPV16-E2 lentivirus
Lenti-Vac: Empty lentivirus
min: Minutes
ml: Milliliters
mM: Millimolar
M-MLV RT: Moloney murine leukemia virus reverse transcriptase
MOI: Multiplicity of infection
NaCl: Sodium chloride
PBS: Phosphate-buffered saline
PCR: Polymerase chain reaction
PE: Phycoerythrin
qPCR: Real-time polymerase chain reaction
RA: Retinoic acid
Rev: Reverse
RIPA: Radioimmunoprecipitation assay
RNA: Ribonucleic acid
rpm: Revolutions per minute
RT: Reverse transcription
SD: Standard deviation
SDS-PAGE: Sodium dodecyl sulfate polyacrylamide gel electrophoresis
μg: Micrograms
μl: Microlites
μM: Micromolar
