# Supplementary material for: HPV16-E2 protein modifies self-renewal and differentiation rate in progenitor cells of human immortalized keratinocytes
Source: Virol J. 2017 Apr 3;14:65. doi: 10.1186/s12985-017-0736-2 (PMC5376701; doi:10.1186/s12985-017-0736-2)
Supplement: Additional file 1: Table S1. — Data from the self-renewal assays. HaCaTwt cells were α6-integrin-CD71 stained and analyzed by flow cytometry to establish the relative abundance of α6-integrinbri/CD71dim (R6), α6-integrinbri/CD71bri (R7) and α6-integrindim (R8) subpopulations (Parental). After sorting, α6-integrinbri/CD71dim or Non-α6-integrinbri/CD71dim cells were re-seeded in separated wells and grown for 10 days. Then, the immunophenotype was analyzed by flow cytometry (First enrichment). Again, α6-integrinbri/CD71dim cells or Non-α6-integrinbri/CD71dim cells were sort and re-seeded for 10 days and flow cytometer analyzed (Second enrichment). Data are presented as the average and SD from three self-renewal assays. (DOCX 13 kb) [file 12985_2017_736_MOESM1_ESM.docx]

**Additional file 1: Table S1. Data from the self-renewal assays**

|  | | α6-integrin^bri^/CD71^dim^ cells | | Non-α6-integrin^bri^/CD71^dim^ cells | |
| --- | --- | --- | --- | --- | --- |
| Subpopulation | Parental | First  enrichment | Second  enrichment | First  enrichment | Second  enrichment |
| α6-integrin^bri^/CD71^dim^  (R6) | **1.16 ± 0.08 %** | **2.80 ± 0.22 %** | **3.76 ± 0.67 %** | **0.17 ± 0.14 %** | **0** |
| α6-integrin^bri^/CD71^bri^  (R7) | 87.27 ± 1.21 % | 84.70 ± 1.4 % | 88.4 ± 1.6 % | 88.51 ± 1.3 % | 56.2 ± 1.3 % |
| α6-integrin^dim^  (R8) | 8.16 ± 0.52 % | 8.53 ± 0.4 % | 8.40 ± 0.58 % | 10.71 ± 0.52 % | 43.79 ± 1.7 % |
